# Mesenchymal Stem/Stromal Cells Microencapsulation for Cell Therapy

**DOI:** 10.3390/cells14030149

**Published:** 2025-01-21

**Authors:** Sharaf Eldeen M. Abbas, Ghada Maged, Hongjun Wang, Ahmed Lotfy

**Affiliations:** 1 Department of Prosthodontics, Faculty of Dentistry, Cairo University, Giza 12613, Egypt; 2 Department of Biochemistry, Faculty of Science, Alexandria University, Alexandria 21526, Egypt; 3 Department of Surgery, Medical University of South Carolina, Charleston, SC 29425, USA; 4 Ralph H. Johnson Veterans Affairs Medical Center, Charleston, SC 29401, USA

**Keywords:** mesenchymal stem/stromal cells, MSCs, microencapsulation, cell therapy, biomaterial

## Abstract

Cell microencapsulation is one of the most studied strategies to overcome the challenges associated with the implementation of mesenchymal stem/stromal cells (MSCs) in vivo. This approach isolates/shields donor MSCs from the host immune system using a semipermeable membrane that allows for the diffusion of gases, nutrients, and therapeutics, but not host immune cells. As a result, microencapsulated MSCs survive and engraft better after infusion, and they can be delivered specifically to the targeted site. Additionally, microencapsulation enables the co-culture of MSCs with different types of cells in a three-dimensional (3D) environment, allowing for better cellular interaction. Alginate, collagen, and cellulose are the most popular materials, and air jet extrusion, microfluidics, and emulsion are the most used techniques for MSC cell encapsulation in the literature. These materials and techniques differ in the size range of the resultant microcapsules and their compatibility with the applied materials. This review discusses various materials and techniques used for the microencapsulation of MSCs. We also shed light on the recent findings in this field, the advantages and drawbacks of using encapsulated MSCs, and the in vivo translation of the microencapsulated MSCs in cell therapy.

## 1. Introduction

Mesenchymal stem/stromal cells (MSCs) are multipotent adult stem cells that can be derived from a variety of mammalian tissues, such as bone marrow, umbilical cord, and adipose tissue, among others [1,2,3]. Their importance originates from their ability to proliferate and differentiate into multiple types of cell lineages. Additionally, studies have shown that MSCs modulate host immunity by secreting immune modulators, including cytokines and growth factors, which could protect them from host immune rejection responses and apoptosis [4,5,6]. These unique characteristics make MSC therapy an attractive approach for cell therapy. However, many challenges still exist upon local or systemic infusion of MSCs, including rapid clearance from circulation, instant blood-mediated inflammatory reaction (IBMIR), and poor homing of exogenous MSC to the targeted sites, which can hamper their therapeutic efficacy [7].

Microencapsulation is a strategy of encapsulating implanted cells with a biomaterial fabricated from natural or synthetic polymers, which can act as a protective shield against the host immune response while allowing essential nutrients to pass to the cells. Hence, they provide a more favorable environment for the implanted cells to survive and grow while avoiding possible fibrosis [8,9]. In addition, microencapsulation is a means of holding and transporting the encapsulated cells to the target bodily site [10]. Major encapsulating materials that have been tested include alginate [11], collagen [12], and cellulose [13], among others.

Recently, investigators have tested the use of encapsulated MSCs as an alternative to traditionally used tissue regenerative medicine [14,15]. In some studies, MSCs were co-cultured with other cell types, including pancreatic islet-derived insulin-producing cells [16] and hepatocytes [17,18] to enhance the growth rate of the cultured cells and to provide higher regeneration capacity. Notably, microencapsulated MSCs hold great promise for tissue regeneration medicine, offering potential treatment for various chronic diseases, including cardiovascular [19] and liver failure [20,21], cancer [22], diabetes [16,23,24], and neurological disorders [25]. In this review, we discuss different materials and techniques used for their microencapsulation and preclinical applications of microencapsulated MSCs in cell therapy.

## 2. MSCs

MSCs are extensively employed in cell therapy due to their many beneficial attributes, such as their potential for self-renewal and differentiation into several lineages without ethical concerns. In addition, MSC therapy is safe, as the risk of teratoma formation and immunogenicity are modest to low [2,3]. MSCs can be isolated from various tissues, including bone marrow, adipose tissue, cord blood, placenta, lung, liver, and skin [4,26]. Consequently, MSCs can differentiate into several tissue forms, including bone, cartilage, muscle, fat, tendon, ligament, and other connective tissues [5]. MSCs must be treated with certain stimuli introduced in a particular sequence for this process to work. In vitro, MSC differentiation is also impacted by the cellular environment, such as hypoxia and inflammatory signals, in addition to the substrate characteristics. For example, it has been demonstrated that rigid culture surfaces promote osteogenesis, while soft gels enhance adipogenesis [27]. In addition, MSCs secrete numerous cytokines and growth factors, including interleukin-2 (IL-2), interleukin-8 (IL-8), monocyte chemotactic protein-1 (MCP-1), stromal-derived factor-1 (SDF-1), vascular endothelial growth factor (VEGF), and transforming growth factor-beta (TGF-β), which regulate the immune system along with numerous intercellular signaling pathways [28,29]. These secreted bioactive compounds can inhibit fibrosis and apoptosis and stimulate organotypic cells, hence boosting their activity [6]. MSCs can, therefore, influence diverse bodily processes and signaling pathways in addition to their differentiation capability.

## 3. Microencapsulation

Various types of biomaterials fabricated from natural or synthetic polymers have been extensively applied to fabricate artificial 3D scaffolds capable of holding, transporting, and protecting cells from the external environment [10,30]. These scaffolds should provide a supportive microenvironment for encapsulated cells that promotes cell survival, proliferation, and the controlled release of therapeutic substances. This has resulted in the development of numerous cell therapy strategies for drug and cell delivery, which are now employed in organ replacement, tissue engineering, and regenerative medicine [8,31,32]. One of these strategies is cell microencapsulation, which aims to overcome the obstacles associated with whole-organ graft rejection and the side-effects of immunomodulatory protocols or immunosuppressive drugs. Cell microencapsulation permits the implantation of both allogeneic and xenogeneic cells while isolating them from the host immune response using a semipermeable membrane that allows the diffusion of gases, nutrients, and therapeutics, but not host immune cells [31].

The scientific community is becoming increasingly interested in this cell-based technology because of its therapeutic potential in many disciplines other than organ replacement. The microencapsulation of cells can be used to release proteins and morphogens over an extended period [9], making it a promising platform for drug delivery. In addition, research has progressed toward designing and constructing active 3D scaffolds that may be used to monitor encapsulated cells [33] or to build biomimetic scaffolds by including peptides in the matrix that influence the destiny of enclosed cells [34]. Cell microencapsulation is considered a cost-effective approach for studying and promoting stem cell growth and differentiation in a 3D environment [14], and an efficient platform for cell retention and delivery in various anatomical sites [15,35]. Here, we discuss various materials and techniques used to microencapsulate MSCs.

## 4. Materials Used for MSC Microencapsulation

To deliver MSCs and preserve their viability and differentiation potential in damaged tissues, it is essential to replicate the in vivo microenvironment using 3D scaffolds. This approach maintains the diverse functions of cells within this 3D environment, including their phenotype, adhesion, metabolism, and response to soluble factors [36]. Furthermore, MSCs interact with other cells more effectively in a 3D environment than a 2D monolayer, enhancing co-culture outcomes and promoting cell growth and tissue regeneration. Microencapsulation is one of the primary techniques for stem cell tissue engineering, which attempts to preserve the survival, phenotype, and differentiation potential of MSCs [15,37]. Furthermore, microcapsules shield MSCs from the host immune response by controlling the exchange of chemical substances between the cells and their environment [38]. In addition, microencapsulation allows MSCs to be co-cultured with other cell types, such as islet cells [16] and hepatocytes [17,18].

Several polymers have been investigated for MSC microencapsulation in research studies. Some of these biomaterials have been tested in clinical settings (see reviews by Trucillo P et al. [39] and Li H et al. [40]); this section discusses the characteristics of these polymers and factors that influence their encapsulation efficiency (Table 1).

### 4.1. Alginate

Alginate is a popular polymer for MSC microencapsulation [11,41,42,43]. This block copolymer comprises subunits of mannuronic acid (M) and guluronic acid (G) [44]. A variety of alginate forms are currently available on the market. Several factors are critical in defining the characteristics and functionality of the polymer, including the M/G ratio, viscosity, purity, and permeability of alginate [42]. For instance, the swelling behavior and stability of microcapsules in the form of core-and-shell structure are highly related to the M/G content. Alginates with a higher G content (lower viscosity) exhibit higher stability and swell at lower rates when compared with high-M-content alginates. Meanwhile, there is no significant difference regarding cell viability or protection against immune cells between both types of alginates [11,41]. On the other hand, it has been reported that high-G-content alginate is less cell-biocompatible than high-M-content alginate, which has a higher swelling tendency that contributes to better cell biocompatibility [42]. Furthermore, the modification of alginate microcapsules by extracellular matrix proteins or their derivatives, such as RGD (Arg-Gly-Asp), can improve the potency of encapsulated cells because these adhesion ligands mimic the natural microenvironment of the engrafted cells [45].

The purity of alginate is also critical for its function as the innate immune system can detect pathogen-associated molecular patterns (PAMPs) in alginate preparations via pattern recognition receptors (PRRs) [46,47], resulting in proinflammatory cytokine production and detrimental anti-capsular immune responses [48]. Toll-like receptors (TLRs) on the cell surface or inside the cells are a type of PRR that recognize PAMPs. Despite rigorous purification, alginate polymers can still contain impurities such as lipopolysaccharides, which are recognized by TLR4 [49], peptidoglycan, and lipoteichoic acid, detected by TLR2 [46], and small molecular poly-M residues, which can be identified by both TLR2 and TLR4 [50]. Therefore, achieving high-quality alginate purification is crucial for ensuring the long-term survival of encapsulated cells.

The permeability of alginate capsules is reported to vary widely in the literature. Some studies indicate that proteins up to 250 kDa and polysaccharides up to 50 kDa can diffuse through alginate capsules [51]. In contrast, other studies suggest that their capsules are impermeable to proteins as small as 25 kDa [52]. This discrepancy may originate from differences in capsule production processes and cell encapsulation techniques, resulting in variations in the capsule’s biochemical properties and pore size. These variations have crucial implications as they affect the effectiveness of the technique, as encapsulated cells can produce immune mediators and respond to host-derived signals [38].

### 4.2. Collagen

Collagens are the most abundant form of animal protein and encompass a family of 28 distinct types. Each collagen type has at least one triple-helical domain. In vertebrates, these collagens are classified using Roman numbers (I–XXVIII). Most collagens are organized into supra-molecular assemblies within the extracellular matrix. Collagens are the most critical component of tissue structures and influence the mechanical characteristics, organization, and shape of the tissue. They regulate cellular processes, including cell proliferation, migration, and differentiation, by interacting with various receptor families [53]. Type 1 collagen is the most widely used collagen in cell microencapsulation due to its exceptional biological qualities to control the development of stem cells. They have been extensively utilized in different applications associated with bone [54,55,56], cartilage [13,57,58], liver [59], and skin regeneration [60].

On the other hand, collagen, like the other polysaccharide hydrogels, has notable drawbacks. For instance, it needs more robust mechanical qualities, and some may be difficult to manage owing to batch-to-batch variance. Polysaccharide hydrogels are frequently mixed with protein-based polymers to create composite or co-polymer hydrogels [15]. Collagen has been introduced into alginate microcapsules as it is a component of the extracellular matrix (ECM) that provides binding sites for cell adhesion to enhance cell proliferation or differentiation [59,60]. The combination of collagen with other polysaccharides, such as agarose [61] and chitosan [54], has also been reported.

### 4.3. Cellulose

Cellulose, a polysaccharide composed of several hundred to several thousand linked D-glucose units in a linear chain, has been explored for MSC microencapsulation [62,63]. Cellulose has many advantages in MSC encapsulation, including its superior biocompatibility, biodegradability, tunable properties, low cost, and renewability [63]. Carboxymethyl cellulose has shown promise in the microencapsulation of bone marrow-derived MSCs (BM-MSCs), with promising outcomes for controlling their osteogenic differentiation [62].

Silanized hydroxypropylmethyl cellulose (Si-HPMC) hydrogel has been used to microencapsulate adipose tissue-derived MSCs (ASCs). The findings demonstrated that Si-HPMC facilitates the nutrient exchange required for the survival of the encapsulated cells [64].

### 4.4. Agarose

In MSC therapy, agarose hydrogels have emerged as versatile materials for delivery, offering a promising platform for creating microenvironments that enhance MSC viability and function owing to their biocompatibility and adjustable mechanical properties. When combined with collagen, agarose has been shown to develop three-dimensional microenvironments that increase MSC survival and retention, and regulate cell development through regulated cell–matrix interactions [61]. Agarose-based hydrogels have also been modified to mitigate host immunological responses by incorporating immunosuppressive substances like Fas ligand (FasL). This strategy has been shown to increase MSC survival in traumatic brain injury models, promoting the expression of neurotrophic factors at the site of injury [65]. Additionally, optimizing agarose–alginate hydrogel formulations has enhanced encapsulation stability and made it easier to transfer viable MSCs, opening up possibilities for broader therapeutic applications [66]. Nevertheless, challenges remain, including limited bioactivity and the need for better degradation profiles. Further investigation has focused on incorporating bioactive components into agarose, which may improve its therapeutic effectiveness in regenerative medicine.

### 4.5. Chitosan

Chitosan-based hydrogels have gained significant interest in regenerative medicine due to their ability to facilitate MSC encapsulation, along with their biocompatibility and biodegradability. These hydrogels provide a biomimetic environment that improves MSC survival and paracrine activity, as well as their therapeutic potential. Injectable chitosan hydrogels, for example, have been used to support MSCs in spinal cord injury treatment. In addition to preserving MSC viability, these hydrogels facilitated the release of MSC-derived vesicles. They maintained their anti-inflammatory and antioxidant qualities, essential for preventing glial scarring and encouraging regeneration in spinal cord injury [67]. Furthermore, adding carboxymethyl chitosan to hydrogels improves MSC osteodifferentiation, enabling early osteogenesis and maturation without external differentiation factors [68]. Additionally, MSCs have been seeded onto macroporous calcium phosphate cement scaffolds using chitosan and β-glycerophosphate (C/GP) hydrogel, which protects the cells during scaffold creation and improves osteoconductivity [69]. These findings demonstrate the versatility of chitosan-based hydrogels as effective MSC delivery vehicles, providing viable options for tissue engineering and regenerative medicine.

### 4.6. Dextran

Hydrogels based on dextran are biomaterials that have attracted attention in tissue engineering and regenerative medicine due to their adaptability. A study used UC-MSCs and oxidized dextran combined with gelatin methacrylate to form injectable hydrogels with excellent biocompatibility and cell delivery capabilities in myocardial infarction treatment. This hydrogel promoted UC-MSC survival, proliferation, and differentiation into cardiac-like cells, exhibiting electrical conductivity similar to that of native heart tissue, which was further enhanced by reduced graphene oxide [70]. Similarly, dextran/gelatin hydrogels loaded with TGF-β3- nanoparticles have shown promise for intervertebral disc degeneration treatment by facilitating MSC differentiation into nucleus pulposus-like cells [71]. Furthermore, dextran-based hydrogels synthesized through thiol–Michael addition reactions, such as Dex-l-DTT, offer adjustable mechanical characteristics and the best 3D microenvironment for encapsulating stem cells while preserving their viability and capacity for differentiation [72]. These diverse applications highlight how dextran can improve cell treatment results, support controlled release mechanisms, and mimic the natural cellular environment, making it an important component in the development of cell-based regenerative therapies.

### 4.7. Gelatin

Gelatin-based hydrogels have become a viable biomaterial in regenerative medicine because they are biocompatible, tunable, and can form MSC-supporting microenvironments. In cardiac repair, gelatin–hydroxyphenyl propionic acid hydrogels have been developed as injectable, in situ cross-linkable carriers for MSCs, significantly improving the survival and retention of encapsulated MSCs [73]. These hydrogels have been shown to reduce fibrosis, enhance myocardial wall thickness, and improve cardiac functions, as evidenced by the improvement in ejection fraction and end-systolic volume. The versatility of gelatin microparticles also allowed them to be integrated into thermally and chemically gelling hydrogels, providing a three-dimensional environment that preserves MSC viability, promotes osteogenic differentiation, and facilitates hydrogel mineralization [74]. Together, these findings highlight the potential of gelatin-based hydrogels in advancing tissue regeneration and cell-based therapies.

### 4.8. Hyaluronic Acid

Hyaluronic acid (HA), a natural glycosaminoglycan, has emerged as a pivotal biomaterial in regenerative medicine and tissue engineering, particularly for MSC applications. Its capacity to mimic natural extracellular matrix enables the creation of sD microenvironments that support MSC survival and development. Compared with conventional 2D cultures, studies have revealed that MSCs grown in HA-based hydrogels exhibit improved proliferation and stemness. For example, alginate–hyaluronic acid (AL-HA) hydrogels upregulate the expression of genes essential for tissue growth and stemness, such as OCT-4, NANOG, and SOX2, while maintaining high survival rates and promoting the formation of cellular spheroids [75]. Additionally, HA hydrogels modified with tyramine and dopamine have demonstrated improved MSC survival under oxidative stress, providing a viable platform for cell encapsulation in harsh microenvironments [76]. The cartilage-mimicking capabilities of HA have also been used for chondrogenesis; MSCs encapsulated in HA hydrogels have outperformed inert materials, like polyethylene glycol, in terms of the significant elevation of cartilage-specific markers, including type-II collagen and aggrecan [77]. These findings underscore HA’s potential as a flexible biomaterial for promoting tailored differentiation, preserving stemness, and advancing MSC administration in various therapeutic applications.

### 4.9. Polyethylene Glycol

Polyethylene glycol (PEG) is a widely used biomaterial in regenerative medicine, often used alone or in combination with other biomaterials to encapsulate MSCs for therapeutic applications. For example, MSCs encapsulated in PEG-collagen hydrogels have been used to deliver their secretome in treating alkali-burn-induced corneal damage. This approach reduces fibrotic repair and restores corneal transparency, suggesting that PEG-based encapsulation may enhance MSC-based therapies for ocular inflammation [78]. In another study, the role of PEG in cell encapsulation was further explored using microfluidic devices to fabricate PEG norbornene (PEGNB) microgels. These microgels, which encapsulated equine MSCs, maintained cell viability for more than 14 days and promoted the secretion of growth factors, such as FGF-2 and TGF-β. Thus, research highlights PEG’s potential to create uniform, supportive environments for MSCs, offering a versatile platform for high-throughput, precise cell therapies in tissue engineering and regenerative medicine [79].

### 4.10. Poly (Lactic-Co-Glycolic Acid)

Poly (lactic-co-glycolic acid) (PLGA) is a versatile biomaterial that has garnered significant attention in tissue engineering and regenerative medicine, primarily due to its ability to encapsulate growth factors and protect therapeutic cells. One study combined PLGA microspheres containing bone morphogenetic protein-2 (BMP-2) and vascular endothelial growth factor (VEGF) with MSCs to reconstruct critical-sized mandibular defects in pigs. The results demonstrated enhanced defect healing, with the PLGA microspheres facilitating the controlled release of growth factors, promoting improved tissue mineralization and bone remodeling [80]. Similarly, PLGA nanoparticles were used to modify MSCs by encapsulating silibinin, a cytoprotective compound. This modification enhanced MSCs’ resistance to oxidative stress, improving their survival and therapeutic efficacy in treating cutaneous wounds [81]. Together, these studies underscore the potential of PLGA as an effective tool in controlled delivery systems for growth factors and cytoprotective agents, significantly enhancing the regenerative capacity of MSCs in challenging clinical applications, like bone repair and wound healing.

## 5. Microencapsulation Techniques

### 5.1. Extrusion

Extrusion is one of the most widely used techniques for cell microencapsulation in regenerative medicine. It includes methods such as electrospray droplet extrusion, air jet extrusion, syringe droplet extrusion, centrifugal extrusion, electrostatic extrusion, and vibrational extrusion. Notably, electrospray droplet extrusion has been extensively applied in stem cell microencapsulation research [36]. This technique, which uses an organic solvent, enables the production of hydrogel beads of approximately 50 µm in diameter without compromising cell viability. The procedure involves gravity dripping, where a hydrogel precursor and cell suspension are extruded through a needle into a hardening solution [63]. The diameter of microspheres is determined by several parameters, including the density of the solution, the diameter of the extrusion needle/nozzle, and the surface tension of the droplets [63]. A homogeneous cell solution and rinsing the nozzle with sodium citrate help to minimize clogging. Additionally, using appropriate settings and a blunt-tip nozzle can help to prevent cell injury [82].

### 5.2. Emulsion

Emulsion-based cell encapsulation typically involves distributing hydrogel precursor in a non-miscible solution, notably a water-in-oil emulsion. At equilibrium, internal gelation occurs, followed by centrifugation to collect the emulsified hydrogels [83]. This method offers various benefits, including lower production costs and scalability. However, the vast size distribution and cell disruptions at the oil–water interface have prompted some concerns. In addition, prolonged exposure to oil and surfactants results in a cytotoxic environment that disrupts cells and adversely affects cell viability [12].

### 5.3. Microfluidics

Microfluidics is a technique for manipulating fluids in microenvironments, enabling the development of microgels. Droplet-based microfluidics represents a potent and versatile tool for reconstructing microenvironments, offering exceptionally high throughput and tight control over cells, biomolecules, and extracellular matrix [84]. There are two major categories of droplet-based microfluidics techniques: active and passive. Passive techniques, such as flow-focusing, co-flow, and T-junction design, are prevalent in cell microencapsulation [85]. In droplet-based microfluidics, droplets are typically produced using flow-focusing and co-flow microfluidic devices, where the shear stress exerted by a continuous phase on a dispersed phase generates the droplets. Typically, both phases are composed of immiscible liquids. In T-junction devices, droplets are formed when two channels meet at the 90° angle and exit by a perpendicular stem. The dimensions of the microchannel and flow rates of the two phases affect the size and shape of the droplets in microfluidics-based synthesis. Owing to the simplicity of droplets and homogeneous size distribution of microbeads, T-junctions are frequently used in microfluidics for cell microencapsulation [86].

### 5.4. Micromolding

Micro-molding is a recently introduced bio-fabrication method for hydrogels with regulated size and shape. Photolithography technology creates these micro-molds with predetermined patterns into which intended polymers are poured and then gelled to create three-dimensional hydrogel constructs [87]. Micro-molding offers several benefits for cell microencapsulation compared with other techniques, including eliminating shear stress from the cell suspension passing through a nozzle and reducing the impact of oils and surfactants that might affect cell viability or behavior [36]. Polydimethylsiloxane was used to create micro-mold chips using photolithography [87] to mitigate osteoarthritis severity. Then, a mixture of adipose-derived stem cells and alginate was poured and gelled inside the micro-mold to form microcapsules of 150 µm size. These micro-encapsulated cells were used for intra-articular injection in a rabbit model. The same research group reported the use of the micro-molding technique to fabricate cross-linked alginate microcapsules with 170 µm size to encapsulate MSCs for better hydrogel stability in the synovial fluid upon its use in intra-articular injection [87].

## 6. Pre-Clinical Studies Using Microencapsulated MSCs

Pre-clinical studies have demonstrated that microencapsulation of MSCs enhances their therapeutic potential in various disease models. This approach improves cell viability, functionality, and immune evasion, making it a promising strategy for regenerative medicine applications across musculoskeletal, cardiovascular, neurological, and other conditions in preclinical settings (Table 2).

### 6.1. Musculoskeletal Diseases

Microencapsulation has been shown to enhance the viability and functionality of MSCs for musculoskeletal tissue regeneration. MSC encapsulation provides a biocompatible 3D microenvironment that facilitates the differentiation capacity of the immobilized stem cells and modulates the inflammatory reaction upon implementation [88,89,90,91,92,93]. For example, when a composite of alginate-microencapsulated rabbit BM-MSCs with β-tricalcium phosphate/calcium phosphate cement was implanted into rabbits with critical size defects, the composite group showed more new bone formation in the bone defects when compared with the control group [94]. Similarly, synchronized 3D vehicle delivery of encapsulated BM-MSCs and the bone morphogenetic protein-2 (BMP-2) showed better efficacy for large bone defects, as evidenced by enhanced osteogenic differentiation and accelerated maturity of newly formed bone tissue [90,95,96].

Additionally, the immobilization of periodontal ligament stem cells (PDLSCs) and gingival MSCs (GMSCs) in alginate microcapsules has been demonstrated as a therapeutically potent strategy in bone tissue engineering [89,97]. Alginate hydrogel microbeads allow the influx of nutrients and oxygen to the encapsulated cells, as well as the efflux of metabolites. This results in prolonged stem cell viability, lasting up to 28 days after encapsulation. The osteogenic differentiation capacity of GMSCs was enhanced by encapsulation, although to a lesser extent in GMSCs. Additionally, ectopic mineralization significantly increases due to both in vitro and in vivo encapsulation, demonstrating their ability to repair calvaria defects [55]. The intra-articular injection of alginate-microencapsulated ASCs was also investigated to manage anterior cruciate ligament transection in a rabbit model. Microencapsulated cells showed a significant chondroprotective effect, which could be a promising strategy for treating osteoarthritis [98].

### 6.2. Cardiovascular Diseases

The use of MSCs as an alternative therapy for cardiovascular diseases is challenging, primarily due to the low retention rate of the introduced stem cells. Studies have reported that less than 10% of cells remain in the heart after one hour of intracoronary infusion [99]. The short retention period may result from various factors, including cell removal through lymphatic or vascular channels or clearance by the immune system.

Encapsulating MSCs before their in vivo application may prolong their initial retention period because the size and physical structure of the capsules protect them from being washed out through lymphatic or venous channels and provide a barrier against the immune system [46]. Bioluminescence imaging has shown that encapsulated MSCs exhibit prolonged retention throughout the myocardium. As a result, encapsulated MSCs reduced scarring after myocardial infarction (MI), significantly enhancing the microvasculature around the area of infarction [99].

Much work has been conducted to elucidate the efficacy and potential of encapsulated MSCs for cardiovascular diseases. Encapsulated MSCs are potent cell therapy approaches for the regeneration of myocardial infarction (MI), as they lead to improved cardiovascular functions and the formation of new blood vessels [99,100]. MSCs encapsulated by arginine-glycine-aspartate (RGD)-coupled alginate are potent in regenerating damaged heart tissue caused by myocardial infarction [101]. The microspheres efficiently enhance the behavior of MSCs and facilitate their transport to the site of damage. They likely serve as a scaffold to preserve the shape of the left ventricle and avoid its adverse remodeling after a myocardial infarction [102]. Furthermore, when MSCs were co-transplanted with Schwan cells, they enhanced angiogenesis inside the ischemic myocardium, resulting in improved cardiac function in rats [103]. Encapsulation in alginate/graphene oxide micro-gel has also been shown to promote MSC antioxidant activity, offering a protective environment against oxidative stress associated with acute myocardial infarction [19]. In comparative studies, encapsulating human MSCs has demonstrated notable superiority over non-encapsulated MSCs in promoting vascular regeneration in a hindlimb ischemia mouse model [104].

### 6.3. Diabetes

The selective destruction of insulin-producing β cells within pancreatic islets is a hallmark of type 1 diabetes, which requires patients to take daily exogenous insulin to maintain normal blood glucose levels [105]. The transplantation of pancreatic islets encapsulated inside alginate microcapsules to avoid islet immune rejection has been considered a treatment option for patients with type 1 diabetes. However, immediate inflammatory reactions around the capsules still occur, leading to the formation of pericapsular fibrotic overgrowth (PFO) and engraftment failure of the islets. PFO occurs because of the host’s inflammatory reaction to antigens released by encapsulated allogeneic or xenogeneic tissue. The formation of a physical barrier, mainly consisting of macrophages and fibroblasts, hinders the transportation of oxygen and other nutrients. Consequently, this results in a state of malnourishment and hypoxia, ultimately leading to the death of the islet. PFO persists despite the use of immunosuppressive therapy. To solve these issues, it is necessary to investigate the co-encapsulation of MSCs with islet cells to utilize their immunomodulating and revascularization potential [23].

The co-encapsulation of MSCs with pancreatic islets has effectively promoted insulin production and maintained normal blood glucose levels in diabetic mouse models [23]. The formation of PFO was dramatically reduced with enhancement in the islet viability, suggesting the immunosuppressive potential of MSCs and their role in improving the functionality of the co-encapsulated islet cells. For further improvements, Razavi and colleagues have developed a non-invasive method in which ultrasound activation is applied for the microcapsules to stimulate both islets and MSCs. This strategy has proven effective in inhibiting islet cell death and maintaining functionality. Furthermore, it has successfully improved the engraftment of islets by promoting their revascularization and mitigating inflammation [106].

### 6.4. Neurological Disorders

MSC therapy has been used as an effective alternative therapy for neurological disorders, mainly based on its immunomodulatory properties via attenuating neuroinflammation and the production of anti-inflammatory cytokines [107]. MSCs have also proven potent in treating central nervous system (CNS) injuries and neurodegenerative disorders, as they can differentiate into neuronal phenotypes [108]. Microencapsulation has been suggested to improve the potency of MSC therapy. For example, encapsulated MSCs have exhibited higher efficacy in mitigating CNS injuries and their associated inflammation [109]. MSCs encapsulated by alginate microencapsulation have effectively attenuated neuroinflammation resulting from post-spinal-cord [110] injury and brain damage [109]. Encapsulated hMSCs not only retain their viability and their secretory activity, but also modulate the inflammatory response. As for the mechanism, encapsulated stem cells secrete anti-inflammatory cytokines, such as IL-4, IL-13, IL-2, IL-1b, and IL-9, upon exposure to pro-inflammatory mediators, such as TNF-α and interferon-gamma (IFN-α). In addition, they regulate the activity of inflammatory macrophages both in vitro and in vivo. This effect was seen even when there was no direct contact between human MSCs and macrophages, promoting the alternative M2 macrophage phenotype [109].

Glucagon-like peptide 1 (GLP-1) has a neuroprotective effect against cytotoxicity and neurodegeneration. Thus, the subcutaneous injection of GLP-1 analogs is an approved therapy for amyotrophic lateral sclerosis (ALS). Alginate-encapsulated MSCs producing GLP-1 have demonstrated effectiveness as a cell therapy for ALS [25]. Encapsulated MSCs have been produced by transfection with a plasmid encoding a GLP-1 fusion gene before injection to improve their neuroprotective properties. Encapsulated GLP-1 MSCs have been injected into an ALS mouse model intracerebroventricularly before disease onset. Animal survival was prolonged by 13 days, the disease onset was delayed by 15 days, and the motor functions were improved; this was associated with reduced serum proinflammatory cytokine levels [25].

### 6.5. Cancer

While MSCs have emerged as a promising cell-based therapy for various cancers, several drawbacks have been identified that may diminish their antitumorigenic potential. For instance, after in vivo infusion, MSCs have been shown to migrate toward tumors and interact directly with tumor stromal elements in the case of colonic adenocarcinoma, multiple myeloma [111], and melanoma [112]. MSC encapsulation has been proposed to provide a 3D microenvironment in which cell contact with cancer cells is inhibited. This approach also has the potential to enhance their therapeutic effects and increase their paracrine activity [22,113].

Alginate-encapsulated MSCs have successfully reduced tumor volume in a syngeneic rat glioma model [113]. The tumor-suppressive effect of encapsulated, unmodified MSCs is about twofold higher than that of MSCs transfected to produce endostatin, an antiangiogenic peptide. Alginate-encapsulated Wharton’s jelly MSCs (WJ-MSCs) are efficient as a cell-based therapy for breast cancer and in inhibiting the formation of the self-renewing cell population, the cancer stem cells [22]. This inhibitory activity was likely attributed to the downregulation of several cancer-associated genes and the induction of cell apoptosis factors, such as caspases, and increased ROS production in cancer cells. In addition, encapsulated stem cells were able to trigger the expression of Wnt antagonists, such as Secreted Frizzled-related protein 4 (sFRP4), Dickkopf-1 (DKK1), and glycogen synthase kinase 3 beta (GSK-3β), downregulating the β-catenin pathway, which is involved in tumor promotion [22].

### 6.6. Liver Diseases

Hepatocytes comprise about 80% of the liver mass and are crucial for liver function. Liver failure is usually associated with hepatocyte malfunctioning [21,114]. Hepatocyte transplantation is an alternative therapy to orthotopic liver transplantation for liver fibrosis and acute liver failure [17,114]. However, some drawbacks have been reported; for example, the immune system initiates a foreign-body-fighting mechanism against the transplanted cells, which hampers its functionality. This was attributed to a possible direct contact between the introduced hepatocytes and recipient immune cells. Immunosuppressive drugs used to attenuate the immune activity can be toxic [59]. Hence, exploring alternative therapeutic strategies, such as microencapsulated MSCs or the co-encapsulation of MSCs with hepatocytes in the recovery of liver failure, has emerged and been investigated [17,20,21,115].

**Table 2:** MSCs and microencapsulation in in vivo models.

Ref.	Cell Type	Encapsulation Material	Encapsulation Technique	Application	Outcomes
[11]	BM-MSCs	Alginate	Syringe droplet extrusion	Osteoarthritis	High G alginate prolonged the presence of metabolically active allogenic MSC in immune-competent rats.
[88]	BM-MSCs	Collagen	Syringe droplet extrusion	Cartilage regeneration	Promotion of chondrogenic differentiation of MSCs when high cell density and high collagen concentration were applied.
[90]	BM-MSCs	Alginate modified with glycine-arginine-glycine-aspartic acid-glycine	Microfluidic	Bone regeneration	Enhancement of osteogenic differentiation and acceleration of mineralization
[91]	BM-MSCs	Fibrin/Alginate	Syringe droplet extrusion	Volumetric muscle loss (VML) injuries	Greater muscle regeneration of rat VML in a shorter period.
[92]	BM-MSCs	Collagen-chitosan	Emulsion	Bone regeneration	Enhancement of ectopic bone formation
[93]	SHED	RGD-modified alginate	Microfluidic	Bone regeneration	Enhanced cell viability and increased ectopic bone formation
[116]	BM-MSCs	Alginate	Electrostatic extrusion	Bone regeneration	Enhanced bone formation and bone marrow growth
[95]	BM-MSCs transduced with BMP2 and/or VEGF	Alginate	Electrostatic extrusion	Bone regeneration	Significant improvement in release of BMP2 and VEGF from genetically modified MSCs with enhancement in osteogenic differentiation.
[96]	BM-MSCs	Alginate	Electrostatic extrusion	Bone regeneration	Promotion of the osteogenic differentiation of BM-MSCs.
[97]	PDLSCs, GMSCs, and BM-MSCs	RGD-modified Alginate loaded with TGF-β3 ligand	Syringe droplet extrusion	Tendon regeneration	Effective differentiation into tendon tissue.
[98]	ASCs	Alginate	Vibrational extrusion	Osteoarthritis	Enhancement of the viability of ASCs in the knee joint and significant reduction in the Osteoarthritis progression and extent.
[117]	BM-MSCs	Alginate	Syringe droplet extrusion	Bone regeneration	Encapsulating MSCs with PEDF improves differentiation and release of cells compared to encapsulation of MSCs alone
[118]	PDLSCs and GMSCs	RGD-modified alginate	Microfluidic	Bone regeneration	Higher amounts of ectopic bone regeneration.
[119]	BM-MSCs	Alginate	Electrostatic extrusion	Orbital bone repair	-Effective induction of osteogenic differentiation. -Greatest bone repair of the orbital wall defect.
[120]	BM-MSCs	Alginate	Electrostatic extrusion	Osteoarthritis	-Chondroprotective effect through paracrine signaling. -Augmentation of the compensatory increases in osteophyte formation.
[121]	PDLSCs and GMSCs	RGD-modified Alginate	Microfluidic	Cartilage regeneration	Chondrogenic differentiation of encapsulated PDLSCs and GMSCs.
[122]	BM-MSCs, PDLSCs, and GMSCs	Alginate	Syringe droplet extrusion	Bone regeneration	Ectopic bone formation around and inside the implemented microcapsules
[13]	BM-MSCs	Alginate–poly-l-lysine–alginate	Emulsion	Enhancement of vascularization	Reduced immune reaction against grafted MSCs cells by microencapsulation
[19]	UC-MSCs	GO/Alginate	Electrostatic extrusion	Myocardial infarction	-Enhancement of the therapeutic activity of the MSCs. -Reduction of post-injection oxidative stress.
[37]	MSCs modified to express erythropoietin	Alginate	Electrostatic extrusion	Erythropoietin delivery	Capsules with lower cell loading showed higher erythropoietin secretion.
[123]	BM-MSCs	Agarose	Syringe droplet extrusion	Vascular regeneration	-Improvement of viability and metabolic activity of the MSCs as well as cell–cytoskeletal patterning. -Significant increase in the number of engrafted cells.
[99]	MSCs	Alginate	Electrostatic extrusion	Myocardial infarction	Higher cell retention and increase in vasculature around infarct site
[101]	MSCs	RGD- modified Alginate	Electrostatic extrusion	Myocardial infarction	-Effective delivery of the MSCs to the site of infraction. -Maintaining the LV shape and preventing its negative remodeling.
[103]	BM-MSCs	Alginate–poly-L-lysine–alginate	Electrostatic extrusion	Myocardial infarction	Angiogenesis augmentation and heart function improvement in acute myocardial infarction.
[104]	BM-MSCs	Alginate	Electrostatic extrusion	Hindlimb ischemia	Significant enhancement of vascular recovery in mouse model of ischemic hindlimb.
[124]	IX–engineered MSCs	Alginate	Electrostatic extrusion	Hemophilia treatment.	-Factor IX secretion was increased by encapsulated MSCs -osteogenic differentiation was also observed
[125]	MSCs	Alginate-poly-L-lysine-alginate	Electrostatic Extrusion	Erythropoietin delivery	Long-lasting (up to 210 days) secretion of erythropoietin after loading the microcapsules in vivo.
[1]	BM-MSCs	Alginate	Air jet extrusion	Islets transplantation	-Improvement of the viability of islets. -MSC–alginate beads exhibited an ability to interactively modulate their microenvironment by IDO activity and secreting several immunomodulatory and trophic factors over a long-term.
[16]	UC-MSCs	Alginate	Syringe droplet extrusion	Type 1 diabetes	Reversal of hyperglycemic status by the synergistic effect of MSCs with pancreatic islet-derived progenitor cells.
[23]	BM-MSCs	Alginate-chitosan	Electrostatic extrusion	Type 1 diabetes	Reduction of blood glucose to levels close to the normal blood glucose level of healthy mice.
[106]	ASCs	Alginate	Air-jet extrusion	Co-encapsulation of MSCs with pancreatic Islets transplantation	Significant improvement in the functionality and viability of the transplanted islets.
[25]	GLP-1 releasing MSCs	Alginate	Extrusion	Amyotrophic lateral sclerosis (ALS)	-Delayed symptom onset and reduction of inflammatory markers -Improved motor performance and prolonged survival.
[110]	MSCs	Alginate poly-L-lysine	Electrostatic extrusion	Post-spinal cord injury	Microencapsulation of MSC is involved in the post-CNS traumatic tissue protective therapy through the conversion of macrophages to the M2 subset.
[126]	BDNF-over-expressing BM-MSCs	Alginate	Air-jet extrusion	Deafness	Improved cochlear implant outcome; increased spiral ganglion neuron survival, bipolar morphology, and neurite outgrowth.
[113]	BM-MSCs	Alginate	Air-jet extrusion	Glioma tumor	Suppression of the tumor growth.
[22]	WJ-MSCs	Alginate	Syringe droplet extrusion	Breast cancer	Down-regulation of pro-proliferation markers, drug transporters, epithelial-mesenchymal transition-associated markers, and angiogenesis-related genes.
[127]	BM-MSCs	Alginate-poly-L-lysine-alginate	Air-jet extrusion	Glioblastoma	A 3-fold decrease in cytokine expression compared to entrapped cell lines.
[17]	BM-MSCs	Alginate-poly-L-lysine-alginate	Electrostatic extrusion	Acute liver failure	Significant enhancement of hepatocyte-specific functions, including albumin secretion and urea synthesis.
[18]	BM-MSCs	Alginate	Air jet extrusion	Pericapsular fibrotic overgrowth around alginate microcapsule PFO	Dose-dependent reduction in PFO and improved graft survival with significantly higher cell viability.
[20]	BM-MSCs	Alginate	Air jet extrusion	Liver fibrosis	Microencapsulated BM-MSCs showed anti-fibrotic effect
[21]	BM-MSCs	Alginate/collagen	Electrostatic extrusion	Liver repair and regeneration	Co-encapsulation with AML12 hepatocytes allowed MSCs to differentiate into hepatocytes and be involved in hepatic regeneration
[35]	ASCs	Alginate	Vibrational extrusion	Cell viability inside the microcapsule	Improved cell viability and retention in vivo.
[128]	BM-MSCs	Alginate	Vibrational extrusion	Cell viability inside the microcapsule	Encapsulated cells remained viable under the kidney capsule with the release of factor bFGF.
[129]	MSCs	Alginate	Extrusion	Cell viability inside the microcapsule	Encapsulated MSCs were active for several weeks and acted as a release system

BM-MSCs (Bone marrow-derived mesenchymal stem cells), SHED (Stem cells from human-exfoliated deciduous teeth), PDLSCs (Periodontal ligament stem cells), GMSCs (Gingiva-derived mesenchymal stem cells), ASC (Adipose-derived stem cells), UC-MSCs (Umbilical cord-derived mesenchymal stem cells), WJ-MSCs (Wharton’s jelly-derived mesenchymal stem cells), BMP2 (Bone morphogenetic protein 2), VEGF (Vascular endothelial growth factor), TGFβ (Transforming growth factor β), BDNF (Brain-derived neurotrophic factor), EPO (Extrapontine), PFO (Pericapsular fibrotic overgrowth), RGD (Arginine–glycine–aspartic acid).

The microencapsulation of MSCs preserved functionality and attenuated immune rejection after MSC transplantation, exhibiting enhancements in liver rescuing [17,20]. The co-encapsulation of hepatocytes and BM-MSCs in alginate-poly-L-lysine-alginate microencapsulation improved hepatocyte-specific functions, including albumin secretion and urea synthesis, both in vitro and in vivo [17]. Thus, the survival rate and liver function were enhanced after the transplantation of encapsulated hepatocytes and MSCs in a rat model of acute liver failure. Furthermore, MSC and AML12 hepatocyte co-encapsulation in volvox spheres promotes hepatocyte regeneration in rat model necrotic liver failure, as assessed by albumin and cytokeratin 18 expression [21]. A volvox sphere microencapsulation comprises a large outer sphere containing smaller spheres that encapsulate the culturing cells, which finally provides a double-layer 3D microencapsulation. The microencapsulation of MSCs by alginate–polyethylene glycol (Alg-PEG) hybrid hydrogel facilitated the protection of MSCs against in vivo immune rejection upon transplantation into the fibrotic liver in mice. Microencapsulated human mesenchymal stem cells decrease liver fibrosis in mice [20]. This protective effect has been attributed to the selective permeability of the hybrid hydrogel, which is permissive to soluble factors, such as oxygen and glucose, and non-permissive to immune cells and antibodies. This property keeps the MSCs from direct contact with the immune cells and mitigates immune rejection after in vivo infusion [20]. All these data support the idea that MSC microencapsulation is a promising cell-based therapy for liver diseases.

## 7. Current Challenges and Future Perspectives

Despite significant advances in MSC microencapsulation, several challenges remain. One notable issue is that the encapsulation process may alter MSC function, including cell survival in the capsule and reducing the secretion of growth factors [38,130]. Additionally, ongoing research is focused on identifying the most effective strategies for utilizing microencapsulated MSCs in regenerative therapies. Numerous studies have investigated factors such as the MSC source, encapsulation materials, delivery mechanisms, and specific pathologies. However, significant gaps and biases persist, which need to be addressed. Furthermore, a method that shows promise for one disease may not yield the same results for others. Further research is needed to optimize encapsulation techniques that preserve and potentially enhance MSC functions for broader therapeutic applications.

One of the unresolved issues is material long-term biocompatibility. The functionality of the capsules is closely related to their interaction with the immune system. The immunogenicity of the capsules triggers a series of cellular reactions, including inflammation, pericapsular fibrosis, and damage to the grafted region. This reaction is harmful to both the subjects and the implanted cells. Multiple strategies have been tested to address this issue. For instance, administering anti-inflammatory molecules such as pentoxifylline [131], dexamethasone [132], and curcumin [133] has shown promising outcomes. Furthermore, the encapsulating polymers themselves might pose a risk for immunogenicity. Polyoxazolines [134] and zwitterionic polymers [135] have low immunogenicity and are more suitable in MSC microencapsulation. These neutrally charged synthetic polymers offer a variety of beneficial qualities over other materials, including high hydrophilicity, minimal non-specific interaction, and minimal immunogenicity [136,137].

Maintaining control over the intra-capsular microenvironment is a significant concern, as cells are not isolated entities, but rather part of a complex and dynamic mixture that includes the cells themselves, extracellular matrix, growth factors, and surrounding cells. Cultivating cells outside their native environments requires a thorough understanding of cell biology. The capsules can be modified with different motifs to replicate their natural habitat. One effective approach involves short peptide sequences comprising functional domains derived from ECM proteins [138]. For example, arginine–glycine–aspartic acid, a component of many extracellular matrix proteins like collagen and fibronectin, has shown promising results [45,139]. Other examples include laminin motifs, such as Tyr-Ile-Gly-Ser-Arg and Ile-Lys-Val-Ala-Val [24]. Moreover, emerging trends in capsule design, such as the development of “smart” biomaterials with real-time monitoring capabilities and stimuli-responsive hydrogels that release therapeutic agents in response to environmental changes (e.g., pH or temperature), promise to address many of the challenges associated with MSC encapsulation.

## 8. Conclusions

**Figure 1:**
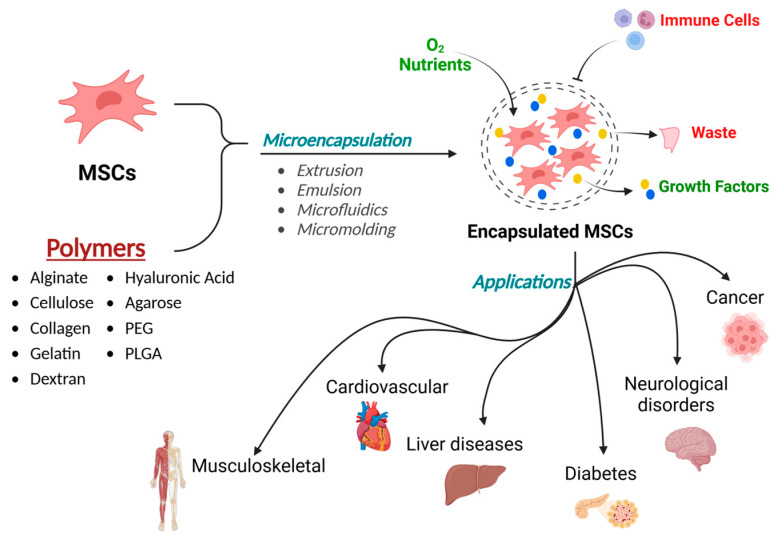
MSCs microencapsulation and their application in different diseases.

This review summarizes studies using microencapsulation techniques to address challenges associated with MSC therapy (Figure 1). A variety of materials and key techniques used for microencapsulation have been explored. This review also highlights the diverse applications of MSC microencapsulation, with a particular emphasis on regenerative medicine and immunomodulation. There is a growing focus on employing microencapsulation technology to effectively replicate the in vivo microenvironment. An ideal microcapsule should provide a 3D scaffold, shield the MSCs from the host’s immune response, and maintain key MSC characteristics, such as phenotypic differentiation and adhesion. Additionally, the microcapsule must exhibit selective permeability, allowing essential nutrients and gasses to pass through while preserving cell viability and preventing fibrosis formation. Achieving this balance is crucial for enhancing the therapeutic potential of MSCs in clinical applications.

## Figures and Tables

**Table 1:** Polymers have been used in MSC microencapsulation and their characteristics.

Material	Biocompatibility	Mechanical Strength	Permeability	Degradation	Immunomodulation	Clinical Challenges	Advantages	Disadvantages
Alginate	High	Moderate	Good	Degrades variably based on cross-linking	Low/moderate	Poor reproducibility and purity standards	Biocompatible, easy to modify	Immunogenic impurities; limited mechanical strength
Gelatin	Moderate	Low	Moderate	Enzymatically degradable	Moderate	Rapid degradation in vivo	Biodegradable, good cell adhesion	Weak mechanical properties
Chitosan	Moderate	Moderate	Low	Slow enzymatic degradation	Moderate	Low solubility at neutral pH	Antimicrobial, supports cell attachment	Solubility issues; moderate biocompatibility
Polyethylene Glycol (PEG)	High	High	Adjustable	Non-degradable or slow (depending on formulation)	Low	Synthetic nature raises regulatory hurdles	Tunable properties, high mechanical strength	Expensive, non-biodegradable
Hyaluronic Acid	High	Low	High	Enzymatically degradable	Low/moderate	Rapid degradation unless chemically modified	Excellent biocompatibility	Poor mechanical strength
Collagen	High	Low	Moderate	Enzymatically degradable	Low	Batch variability and weak mechanical properties	Excellent biocompatibility, natural ECM mimic	Limited stability
PLGA (Poly (lactic-co-glycolic acid))	High	High	Adjustable	Degrades via hydrolysis into lactic and glycolic acid	Low	Potential acid accumulation causing inflammation	Tunable degradation rate	Expensive, inflammatory degradation products
Agarose	Moderate	Moderate	Low	Non-degradable	Low	Limited mechanical tunability	Easy to handle, good thermal stability	Non-biodegradable, limited cell adhesion
Cellulose	High	Moderate	Moderate	Non-degradable or slowly enzymatic	Low	Limited modification options for specific applications	Abundant, biocompatible, supports cell adhesion	Poor biodegradability, difficult to process
Dextran	High	Low	High	Rapid enzymatic degradation	Low	Rapid degradation unless chemically modified	Excellent biocompatibility, easy to functionalize	Weak mechanical properties, short in vivo stability

## Data Availability

Not applicable.
